# The Sterile Insect Technique for Controlling Populations of *Aedes albopictus* (Diptera: Culicidae) on Reunion Island: Mating Vigour of Sterilized Males

**DOI:** 10.1371/journal.pone.0049414

**Published:** 2012-11-21

**Authors:** Clelia F. Oliva, Maxime Jacquet, Jeremie Gilles, Guy Lemperiere, Pierre-Olivier Maquart, Serge Quilici, François Schooneman, Marc J. B. Vreysen, Sebastien Boyer

**Affiliations:** 1 Maladies Infectieuses et Vecteurs Ecologie, Génétique, Evolution et Contrôle (IRD 224-CNRS 5290-UM1-UM2), Montpellier, France; 2 Centre de Recherche et de Veille sur les Maladies Emergentes dans l’Océan Indien, Sainte Clotilde, La Réunion, France; 3 Insect Pest Control Laboratory, Joint FAO/IAEA Division of Nuclear Techniques in Food, International Atomic Energy Agency, Vienna, Austria; 4 Etablissement Français du Sang, Saint Denis, La Réunion, France; 5 Cirad, UMR PVBMT Cirad/Université de La Réunion, Pôle de Protection des Plantes, Saint-Pierre, La Réunion, France; National Institute for Communicable Diseases/NHLS, South Africa

## Abstract

Reunion Island suffers from high densities of the chikungunya and dengue vector *Aedes albopictus*. The sterile insect technique (SIT) offers a promising strategy for mosquito-borne diseases prevention and control. For such a strategy to be effective, sterile males need to be competitive enough to fulfil their intended function by reducing wild mosquito populations *in natura*. We studied the effect of irradiation on sexual maturation and mating success of males, and compared the sexual competitiveness of sterile versus wild males in the presence of wild females in semi-field conditions. For all untreated or sterile males, sexual maturation was completed within 13 to 20 h post-emergence and some males were able to inseminate females when 15 h old. In the absence of competition, untreated and sterile males were able to inseminate the same number of virgin females during 48 h, in small laboratory cages: an average of 93% of females was inseminated no matter the treatment, the age of males, and the sex ratio. Daily mating success of single sterile males followed the same pattern as for untreated ones, although they inseminated significantly fewer females after the ninth day. The competitiveness index of sterile males in semi-field conditions was only 0.14 when they were released at 1-day old, but improved to 0.53 when the release occurred after a 5-day period in laboratory conditions. In SIT simulation experiments, a 5∶1 sterile to wild male ratio allowed a two-fold reduction of the wild population’s fertility. This suggests that sterile males could be sufficiently competitive to mate with wild females within the framework of an SIT component as part of an AW-IPM programme for suppressing a wild population of *Ae. albopictus* in Reunion Island. It will be of interest to minimise the pre-release period in controlled conditions to ensure a good competitiveness without increasing mass rearing costs.

## Introduction

In Reunion Island (21°10” S; 55°30” E), *Aedes albopictus* Skuse can be found at very high densities and its habitat extends from urban areas to inhabited ravines [1]. Its wide distribution had a high impact on chikungunya disease transmission and thus leads to major health issues; a third of the Reunion human population was infected by the chikungunya virus during the 2005–2006 outbreak [2]. Research is being conducted to assess the feasibility of including the sterile insect technique (SIT) in an area-wide integrated pest management (AW-IPM) program targeting this species [3].

*Ae. albopictus* shows an efficient adaptative behaviour in Reunion Island as it can be found in various habitats [4]. As predation pressure cannot regulate its densities, the most limiting density-dependent effect on the population should be due to intra- or inter-specific competition [5]. Reproductive competition through the SIT could be an additional powerful tool to control populations of this pest: successive releases of sterile males would allow reducing the number of offspring in the following generations and may help controlling the density of this species in urban areas where it threatens the health of human populations [6]. Encouraging results were reported from a pilot trial during which sterile male *Ae. albopictus* were released in Northern Italy [7].

The sexual competitiveness of the released sterile males could be a major limitation for the use of SIT to control mosquito populations. The competitiveness value of males may be linked to various parameters such as their survival, their aptitude to find females, mate and transfer semen and the number of females that a male is able to copulate with. Each of these parameters might be affected during the sterilization process. Irradiation causes random dominant lethal mutations in the germinal cells resulting in the death of the developing embryos after fertilization [8], but depending on the radiation dose and the stage of development of the insect, this process might affect somatic cells as well. Somatic damage is usually visible through a reduction of the longevity, sexual vigour and general activity of males [9]. This is a particularly important point of research for the success of the SIT, as the released male’s ability to find a female, mate and successfully transfer their sterile sperm must not be reduced in order to achieve highest efficiency. It is recommended to irradiate males as late as possible in the life stage as the sensitivity to radiation decreases because by then most of the cells would have finished their mitotic division [10], [11]. The effect of radiation on insect pupae is higher than on adults, the latter generally leading to a sterile male competing almost equally with wild males [12], [13]. Practical reasons however encourage the irradiation at the pupal stage for mosquito as handling of pupae is easier and adults are more fragile.

In addition to the irradiation process that can affect competitiveness [8], the rearing history of the colony and the lack of genetic diversity induced by the laboratory colonisation might also alter male’s sexual vigour under field conditions [14]. Males adapted to mate in a confined space might not be able to behave as wild males under field conditions. Moreover, assortative mating with laboratory-reared females of a similar genetic background might result in a good competitiveness but would not necessarily reflect mating with wild females [15], [16], [17], [18]. This emphasizes the importance of determining the competitiveness in semi-natural conditions with females and males coming from wild larvae or eggs.

The purpose of this research was to assess the effect of irradiation on various parameters of sterile male reproductive value, in the context of the development of the SIT for the control of *Ae. albopictus* in Reunion Island. We compared the mating success between sterile and untreated males in laboratory conditions and we performed competitiveness tests against wild specimens under semi-field conditions.

## Materials and Methods

### Ethics Statement

According to the French legislation (decree 2001-464 from May 29, 2001) experiments carried out on invertebrates are not considered as animal testing and hence are not subjected to regulation. Similarly, as no experimentations were carried out on mice and rabbits, they are not subjected to this regulation; their use concerned only the blood-feeding of the mosquitoes for which purpose one mouse was placed inside the mosquito cage for 20 min. Their rearing strictly followed the Guide for the Care and Use of laboratory animals [19] and was approved by the Institutional Animal Care and Use Committee, Veterinary Services Direction, Saint Pierre, Reunion Island, as well as their use in the described experiments. A daily monitoring ensured their wellness, and none of them were sacrificed for the experiments. Mice were kept three per cage (28×18×15 cm) and fed with hay pellets (Compagnie des Grains du Capricorne, Le Port, Reunion Island); rabbits were reared in pens (200×l55×100 cm) but kept one per cage (100×l55×44 cm) during three consecutive nights for the semi-field experiments, they were fed with rabbit food and occasionally fresh vegetables. The director of Cirad La Bretagne permitted the use of the land for the location of the semi-field cages. These semi-field studies did not involve any endangered or protected species. Human volunteers were part of the team and of the co-authors. The research protocols are approved by the Comité Consultatif de Déontologie et d’Ethique (CCDE) of the research center IRD.

### Mosquito Stocks and Rearing

The strain of *Ae. albopictus* used in the experiments (excluding the wild adults used in the competitiveness tests) originated from Saint-Benoît, Reunion Island. Immature and adult mosquitoes were reared in a climate-controlled room maintained at a temperature of 27±2°C and 75±2% relative humidity; the light regime was LD 12∶12 h photoperiod. Generations F3 and F4 were used for this study. Egg hatching was triggered using a highly dehydrated rabbit food left overnight in the rearing water (haypellet, Compagnie des Grains du Capricorne, Le Port, Reunion Island). Larvae were reared at a density of approximately 500 first instar larvae (L1) per tray (30×40 cm) containing 1 litre of water. They were fed with rabbit-food and fish-food (Sera Koi Food, Sera, Heinsberg, Germany). Pupae were collected and placed in small plastic cups inside an insect adult cage (30×30×30 cm) for emergence. Adults were continuously supplied with 10% sucrose solution [w/v]. Females were blood-fed on mice and eggs were kept at room temperature after a three-day period of maturation.

### Irradiation Procedure

Pupae from the Saint-Benoît laboratory strain were collected less than 9 h after the formation of the first pupa and were irradiated when 24 to 30 h old. They were maintained in cups of 4 cm diameter filled with water at the centre of the irradiation chamber. They were exposed to gamma rays emitted by a cesium-137 irradiator (IBL 437, Cis Bio International, Germany); the dose rate was ca 2.35 Gy/min. In the text non-irradiated males are referred to as untreated males.

### Sterility Curve

Male pupae were irradiated at 0, 15, 25, 35 and 40 Gy. After emergence 70 males were caged with 35 virgin females. After five days, females were blood-fed on mice and allowed to lay eggs in individual tubes. Eclosion rate of each egg batch was then recorded to assess the mean fertility. According to the results, it was decided to use the sterilizing dose of 40 Gy for mating success experiments. However, the irradiator available was owned by the hospital and the equipment set to deliver a dose of 35 Gy for blood irradiation. As each modification of the radiation dose required a time-consuming and complex handling process, we decided to use a dose of 35 Gy for the competitiveness tests, which required frequent use of the source, as a convenient alternative for the operators.

### Recovery of Fertility

Nine experiments were conducted in which one 40 Gy-irradiated male was allowed to mate with 2- to 3-day-old virgin females during two periods of five days separated by a five-days resting period without females. A different group of 2- to 3-day-old virgin females was used for the male’s second mating period. Females were blood-fed on a human volunteer arm and allowed to lay eggs in individual tubes; egg hatch rates were recorded.

### Effect of Irradiation on Male Sexual Maturation

The stage of genitalia rotation [20] was recorded every hour from 0 to 30 h post-emergence for untreated and 35 Gy irradiated males. For each time point, a different group of males was frozen and observed under a binocular microscope. The following divisional markers were used to categorize five stages of the rotation of the male genitalia: stage 0, no rotation; stage 1, ≤45° rotation; stage 2, >45°–≤90° rotation; stage 3, >90°–≤135° rotation and stage 4, >135° to complete rotation of 180° [21]. The rotational direction (clockwise or counter-clockwise) was also recorded.

Ten males emerging within 30 min were immediately caged with 10 females (4- to 5-day-old) for 15, 20 or 25 h. All adults were then frozen. Females were dissected and the three sperm storage organs (spermathecae) were observed in order to determine how many were inseminated. Male terminalia rotation stage was also recorded. For both untreated and 35-Gy-irradiated males, five replicates were carried out for 15 h old males and ten replicates were carried out for 20 and 25 h old males; two different cohorts were used. Females were frozen 2 h after the end of the mating period to give sufficient time for the transferred sperm to be stored in the spermathecae [22].

### Insemination Rates

Small laboratory cages (30×30×30 cm) were filled with 50 one- or five-day old untreated males and 50 virgin females. The same cages were set up with males sterilized at 35 Gy. A last cage contained 250 one-day old sterile males together with 50 females. After 48 h, the females were dissected and the spermathecae were observed to determine the insemination status.

### Daily Mating Success

One male was caged with 10 virgin females (2- to 4-day-old) for 15 days. Every 24 h, females were removed, frozen, and replaced by 10 virgin females (2- to 4-day-old). They were then dissected and checked for spermathecae insemination status. Wing lengths were also measured. The experiment was replicated five times for both untreated males and 40-Gy-irradiated males.

### Effect of the Age on Mating Success of Sterile Males

Ten experiments were performed in which one sterile male (irradiated at 40 Gy) was left together with 10 virgin females (2- to 4-day-old) for 5 days. In a first group, males were one-day old at the beginning of the experiment, and five-days old in the second group. After the mating period, females were dissected and checked for spermathecae insemination status.

### Competitiveness of Sterile Males against Wild Males

Three semi-field cages (6×3×2 m) were set up on gravel soil bordered with tall trees providing shadow. A table was placed in the cage to offer shelter to the insects. Three cups containing cotton dipped into a 10% sugar solution were surrounded with water to prevent the access of ants and honey drops were put into water to attract the mosquitoes towards the sugar sources. In order to render the experimental conditions as restrictive as possible for the sterile males, wild males and wild females originated from eggs collected in the field close to the semi-field cages, and were therefore already adapted to the local environmental conditions. Laboratory males were irradiated as pupae at 35 Gy as described above. Larvae were reared under the same conditions as for the laboratory colony. Males and females were released at the same time in the cage and a rabbit was left for three days in a small cage under the table to provide the necessary blood source for females. On the fourth day, the rabbit was removed and four oviposition cups were placed in each semi-field cage for three nights. Egg cups were removed on the seventh day and the remaining mosquitoes were trapped using BG-traps [23]. This experimental set up did not allow females to undergo two gonotrophic cycles as they did not have the possibility to blood-feed after oviposition. Thus all the egg batches collected on the 7^th^ day were laid by a different female. Eggs were maturated for three days and hatched under laboratory conditions. Hatching rates and numbers of larvae were recorded.

Three different conditions were tested: (experiment 1) ratio 1∶1:1 (1 sterile male: 1 wild male: 1 female) with one-day-old adults, (experiment 2) 1∶1:1 with five-days-old adults and (experiment 3) ratio 5∶1:1 with one-day-old adults. When one-day-old adults were released, pupae were placed in a small cage inside the semi-field cage for emergence and males and females were released on the following morning. When 5-days-old adults were released, emergence occurred in the insectarium; males and females were kept separately and fed under laboratory conditions for five days before the release. When the release ratio was 1∶1:1, 200 sterile males, 200 wild males and 200 wild females were released whereas when the ratio was 5∶1:1, 500 sterile males, 100 wild males and 100 wild females were released. Experiments 1 and 2 were replicated 6 times, and experiment 3 was replicated 5 times. Controls for the fertility levels of wild and irradiated males were carried out under laboratory conditions.

The competitiveness index (C) was calculated as ((Hn-Ho)/(Ho-Hs))*(N/S); where Hn and Hs were respectively the hatch rate from eggs of females mated with untreated or sterile males, Ho was the observed egg hatch rate in the experiment and N and S were the numbers of untreated and sterile males respectively [24]. The variance of C was calculated according to Hooper & Horton [25].

### Statistical Analysis

Shapiro and Bartlett tests were performed to test respectively the normality and the homoscedasticity of the data.

For the sterility curve experiment, differences between egg hatch rates as a function of radiation dose were analysed using a one-way ANOVA after a square-root transformation of the data. In the recovery of fertility study, the fertility rates of females mated by irradiated males was compared during the first or second mating period using a two-tailed paired Student’s t-test after square-root transformation of the data. For the sexual maturation study, proportion tests with continuity correction were used to compare the paired proportions of untreated versus sterile males for a given rotation stage and a given time; one-way ANOVA were used to compare the mean number of females inseminated after a given period. For the insemination rate study, the proportions of females inseminated by males in the different situations were compared using proportion tests with continuity correction. For the mating success tests, the mean number of females and inseminated spermathecae per female was compared between untreated and sterile males, as well as between young and old sterile males, using one-way ANOVA. A two-tailed paired Student’s t-test was used to compare the number of females inseminated for a given number of days between untreated and sterile males; and proportion tests with continuity correction were used to compare the proportion of females with 1, 2 or 3 spermathecae filled. Logistic regression was used to test the effect of female wing size on their inseminating status.

For all the tests, the alpha level was *P*<0.05. The dataset was analysed using R software [26]. Values in the text are expressed as mean ± SEM.

## Results

### Sterility Curve

**Figure 1:**
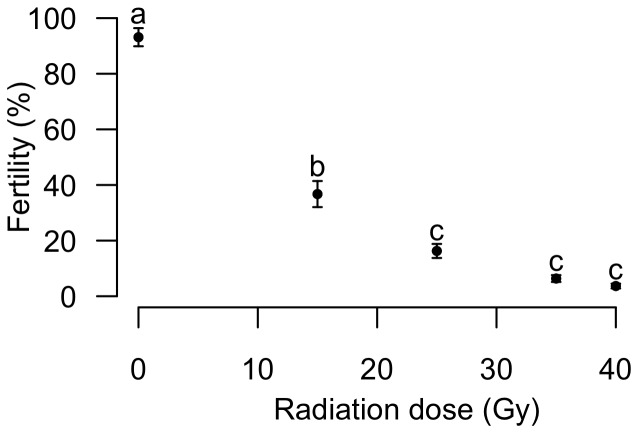
Sterility curve of male *Ae. albopictus* from Reunion Island. Mean fertility (%) as a function of radiation dose. Unlike letters indicate significant difference between the points (*P*<0.05).

Male *Ae. albopictus* from the Reunion strain were exposed to gamma radiation at various doses and crossed with non treated females. The control fertility was 97%. It was reduced to 7% and 4% by irradiation with 35 and 40 Gy, respectively (Fig. 1). As no significant difference existed between the sterility levels induced by these two radiation doses, the 35 Gy dose was used for the competitiveness experiments.

### Recovery of Fertility

The persistence of male sterility after irradiation was tested. In this experiment, females mated with 40 Gy irradiated males aged 1 to 5 days had a mean fertility of 3.0±0.1%, which was not different from the result of the sterility curve. Each male inseminated 1 to 4 females during this first mating period. During the second period of mating, the same males then aged 10 to 15 days old were able to inseminate 1 to 6 females; the fertility of all of these females (N = 17) was zero, which was significantly lower than during the first period (two-tailed paired Student’s t-test, t = 6.32, df = 21, *P*<0.001). During these two 5-days periods an individual male was able to inseminate a total of 2 to 8 females.

### Effect of Irradiation on Male Sexual Maturation

The temporal sexual maturation process of freshly emerged untreated and sterile males was assessed by examining the terminalia rotation and the insemination ability.

Terminalia rotation could be detected 4 h post-emergence for untreated males. After 10 h, all males had started the rotation and the first males with the terminalia fully rotated were observed after 11 h (25%). After 17 h, all males were in stage 3 or 4. In groups aged 18 to 25 h old, 80 to 100% of the males had completed rotation.

The first irradiated males recorded in stage 1 were observed 3 h after their emergence, which was significantly earlier than for untreated males (proportion test with Yates correction, *X^2^* = 4.02, df = 1, *P*<0.05). Similarly to the untreated males, after 10 h every sterile male had started the rotation. However, a delay in the rotation speed was observed in sterile males aged 15 to 19 h compared with untreated ones. The first fully rotated sterile males were observed after 13 h (11%) and the rotation process was completed 20 h post-emergence. Significant differences between untreated and sterile males were observed at the age of 12 h (*X^2^* = 1.56, df = 1, *P*<0.01), 15 h (*X^2^* = 9.77, df = 1, *P*<0.01), 16 h (*X^2^* = 12.8, df = 1, *P*<0.001), and 18 h old (*X^2^* = 6.89, df = 1, *P*<0.01).

**Figure 2:**
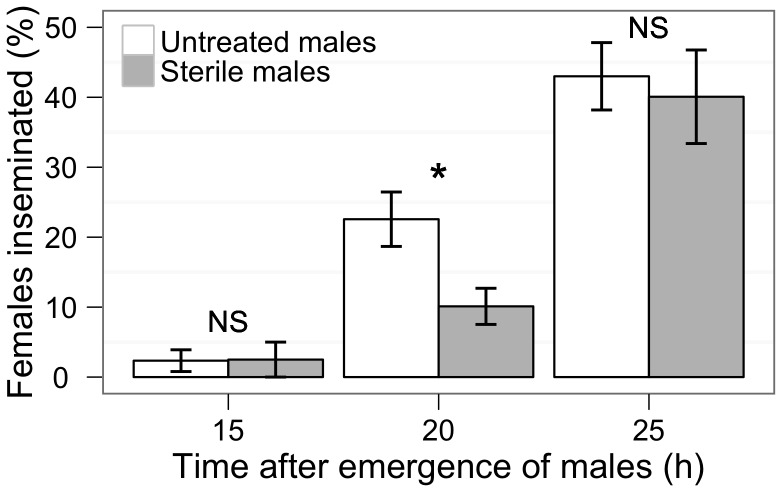
Insemination ability according to age and sterilization for male *Ae. albopictus*. Percentage (± SEM) of females inseminated in small cages (10 females and 10 males) for different durations after male emergence (3 replicates). NS indicates a non-significant difference and * stands for a significant difference (*P*<0.05) between sterile and untreated males.

Very few males were able to inseminate females during the first 15 h of their adult life as only 1 cage out of 5 contained a female inseminated for both untreated and sterile groups. After 20 h post-emergence, untreated males had inseminated twice as many females as sterile males (one-way ANOVA, F_(1, 23)_ = 5.66, *P*<0.05; Fig. 2). However after 25 h, all the cages contained inseminated females and both untreated and sterile males had a comparable insemination capacity with respectively 43 and 40% of females being inseminated: 87% of these females had 2 spermathecae out of three filled with sperm.

### Insemination Rates

The insemination rate of a group of 50 females caged for 48 h with 1- or 5-day old untreated or sterile males, at a 1∶1:1 or 5∶1:1 ratio was assessed. No statistical difference in insemination rates was observed in relation to age, irradiation treatment or sex ratio (Table 1): on average 93% females were inseminated after 48 h, and 92% of them had 2 filled spermathecae.

**Table 1:** Insemination rates of untreated or sterile male *Ae. albopictus* (50 females, 48 h).

Male	Age (days) at release	Male:Female ratio	Number of spermathecae inseminated (% of females)			
			0	1	2	3
Untreated	1	1∶1	2.0	0.0	91.8	6.1
Sterile	1	1∶1	12.2	6.1	81.6	0.0
Untreated	5	1∶1	6.5	4.3	84.8	4.3
Sterile	5	1∶1	6.5	8.7	80.4	4.3
Sterile	1	5∶1	6.4	0.0	89.4	4.3

Percentage of females with 0, 1, 2 or 3 spermathecae inseminated in laboratory cages in the different situations of the competitiveness tests.

### Daily Mating Success

In order to assess male mating performance, a new group of 10 females was offered daily to one sterile or untreated male for 15 days.

Over the 15-day period, sterile males inseminated significantly fewer females (one-way ANOVA, F_(1, 104)_ = 4.89, *P*<0.05) per day compared with untreated males (Table 2). On the first day, a fertile male inseminated on average twice as many females than a sterile one, but this difference was not statistically significant (two-tailed paired Student’s t-test, t = 2.78, df = 4, *P* = 0.07). The number of females inseminated per day decreased until the 5^th^ day and increased again during the next 5 days; this cyclic pattern was observed for both untreated and sterile males. The mean number of females inseminated by a sterile male was always slightly lower than for an untreated male, but this difference was significant only after the 9^th^ day (t = 2.36, df = 7, *P*<0.05). Most of the inseminated females had only one spermatheca filled, only 18 and 6% of them had two spermathecae filled when mated by untreated and sterile males respectively; this difference was not significant (proportion test with Yates correction, *X^2^* = 2.98, df = 1, *P* = 0.084). Female wing size was not correlated to the insemination status (logistic regression, *z* = 1.56, df = 659, *P* = 0.16 for untreated males; *z* = −0.176, df = 509, *P* = 0.86 for sterile males).

**Table 2:** Mating success of untreated or sterile male *Ae. albopictus*.

	Untreated males		Sterile males		Statistical difference
	Mean	SD	Mean	SD	
N inseminated females/male/day	1.5	0.5	1.0	0.3	*
N spermathecae inseminated per female/male/day	1.8	0.5	1.1	0.3	**
N inseminated females after:					
1 day	2.4	0.5	1.2	0.8	NS
5 days	8.2	1.1	6.8	1.6	NS
9 days	14.6	3.8	9.5	1.3	*
14 days	19.2	4.1	12.0	–	–

Mean number of females inseminated per day, spermathecae filled per female per day, and cumulative number of females inseminated over a certain period, per male. Ten new females were given every day (5 replicates). NS indicates a non significant difference, *and **stand for a significant difference between sterile and untreated males at *P*<0.05 and *P*<0.01 respectively.

### Effect of the Age on Mating Success of Sterile Males

In order to compare the mating performance of 1- and 5-day old sterile males, the number of females inseminated by one male after 5 days was assessed.

There was no significant difference between young and older sterile males for the number of inseminated females (one-way ANOVA, F_(1,17)_ = 0.95, *P* = 0.34) or spermathecae filled per female (F_(1,17)_ = 1.82, *P* = 0.2). Sterile males aged 1 to 5 days old inseminated on average 3.3±0.5 females of which 15% and 67% had respectively one and two spermathecae filled. When aged 5 to 10 days old they inseminated on average 2.7±0.4 females of which 17% and 79% had respectively one and two spermathecae filled.

### Competitiveness of Sterile Males against Wild Males

Experiments were carried out under semi-field condition to assess the fertility of the caged population and the competitiveness index of sterile males when they where competing with wild males for inseminating wild females; adults were released at 1- or 5-day old and in a 1∶1:1 or 5∶1:1 ratio.

The mean fertility of the wild males used for these experiments was 93±1% and 5±0.9% for males irradiated with 35 Gy. Preliminary tests in non-competitive situations were conducted to ensure that both wild and irradiated males were able to survive and to inseminate wild females in this experimental set-up under semi-field conditions.

In competitive situations, when males were released in the cage on the day following their emergence, with an equal ratio of wild and sterile males, the mean fertility was reduced to 82.7% (Table 3), which was significantly higher than for the two other experiments (one-way ANOVA, F_(2,14)_ = 17.7, *P*<0.001); this resulted in competitiveness indexes (C) ranging between 0.06 and 0.35. When males and females were kept 5 days under laboratory conditions before the release in the semi-field cages, the mean fertility was reduced to 62.2%, C values ranged between 0.44 and 0.76, and were significantly different from the two other experiments (F_(2,14)_ = 9.71, *P<*0.01). When increasing the ratio of 1-day-old sterile to wild males, the mean fertility was twice as low as the one of a wild population as it averaged 46.4±7.6%. High variations of fertility and thus C were observed between the five replicates in experiment 3, C varying between 0.10 and 0.62.

**Table 3:** Competitiveness indices of sterile male *Ae. albopictus* under semi-field conditions.

	Conditions		
	Ratio 1∶1:1	Ratio 1∶1:1	Ratio 5∶1:1
	1 d old	5 d old	1 d old
N replicates	6	6	5
N adults released	200∶200:200	200∶200:200	500∶100:100
Mean fecundity*(±SEM)	5395±695 a	7685±737 b	2339±406 c
Mean % fertility(±SEM)	82.7±2.8 a	62.2±1.5 b	46.4±7.6 b
Mean C (± SEM)	0.14±0.14 a	0.55±0.12 b	0.23±0.19 ab

Mean fecundity and fertility values of wild females in the different conditions (ratio of sterile male: wild male: wild female and age at release varied) and competitiveness index (C) of sterile males. C was based on the fertility levels of control wild males (93.5±0.4%) and sterile males (4.9±0.3%). Unlike letters indicate significant difference between the row values (*P*<0.05).

* Number of eggs collected in one semi-field cage.

The mean fecundity differed significantly between each experiment (F_(2,14)_ = 17.37, *P*<0.001). According to the previous experiment, a female oviposited an average of 50 eggs. If this assumption is correct, the mean number of females participating in the egg laying was 108, 154, and 47 respectively for the experiments 1 to 3. In experiment 1 and 2, 200 females were released whereas only 100 were used for the third test; therefore half of the females would have laid eggs in experiments 1 and 3, and 75% of them in experiment 2. The age of the females during the whole test differed between these experiments, as they were aged 1 to 7 days old in experiments 1 and 3, and 5 to 12 days old in experiment 2.

## Discussion

The ^137^Cesium irradiation of pupae from Saint Benoit (Reunion Island) strain of *Ae. albopictus* males induced a similar decrease of fertility levels as reported by Balestrino et al. [27] on the Rimini (Italy) strain of the same species. The sterility induced by a 40 Gy irradiation was permanent as successive matings and a resting period did not show any recovery of fertility in males. The fertility was even reduced, as all the females inseminated during the second mating period were completely sterile. Radiation damage is higher on the earlier stages of spermatogenesis than on mature cells [28]; assuming that the sperm used during the second mating period originated from sperm cells that were immature during the irradiation process, and hence more radiosensitive, it is likely that this would result in the total sterility of the sperm cells. Patterson et al. [29] also showed permanent sterility in radio-sterilized males *Cx. quinquefasciatus* Say during 2 weeks. However, our results are different from those obtained in Italy with the Rimini strain where male *Ae. albopictus* showed a slight but non significant increase in fertility with male age [30].

Sterile male mating vigour as shown by the mating success tests was not significantly affected during the first week of adulthood, but the males became less efficient thereafter. Although the male mosquitoes inseminated fewer females each day, the differences were significant only from day 9 onwards. After that period, untreated males inseminated 50% more females than sterile ones. As the irradiation may interfere with the maturation of new sperm cells, it seems likely that sterile males might have fewer successful matings (i.e. leading to insemination), therefore a lower daily mating success might be expected after several matings. However, using a 1∶5 male-female ratio during 4 h, Balestrino *et al*. [27] observed no effect of a 40 Gy irradiation on the mating performances of male *Ae. albopictus*, but they reported fewer successful matings for 50-Gy-irradiated males. Grover *et al*. [31] reported that chemosterilization slightly reduced the insemination rates of male *Ae. aegypti* for periods of 24 h or 48 h over 10 days; on the other hand, radiosterilized males *An. pharoensis* Theoblad [32] or ***C*** *x. Quinquefasciatus* Say [29] did not show a different mating success compared with laboratory males.

We observed that when sterile and untreated males were offered new receptive females daily, creating intensive mating opportunities, most of the females had only one spermatheca filled. This pattern was also reported by Boyer *et al.* [33] for untreated male *Ae. albopictus* from another strain of Reunion Island. However, we showed that when a male was enclosed with the same 10 females for a longer period or when several males and females were caged together, females had mostly two spermathecae filled. We hypothesized that males from this strain might inseminate most of the time only one spermatheca per mating attempt and when kept with the same females for several days they might have more opportunities to transfer more sperm to the same female thus filling two spermathecae. The filling of only one spermatheca should not prevent female egg laying. Contrary to anopheline [34], the oviposition behaviour in *Ae. albopictus* and *Ae. aegypti* females would not be dependent on the spermathecae containing sperm but would rather be triggered by proteins from the male accessory gland (MAG) secretion that are transferred together with the sperm in the *bursa inseminalis* [35], [36], [37]. In addition, as the MAG substance would diminish the female propensity to be inseminated by another male [22], a female with only one spermatheca filled should therefore not have a higher probability of multiple-insemination. However, this would need further investigation as recent studies indicated the occurrence of multiple matings in the field for *Ae. albopictus* [38] and in semi-field condition for *Ae. aegypti* [39]. In the case of the SIT, multiple inseminations would not be prejudicial if sterile and wild males are equally involved, and when the sterile males outnumber the wild ones. The conditions and duration of mating success tests in laboratory might affect the possibility to highlight differences or not between sterile and untreated males, while carrying out these tests in a semi-field environment can help getting a realistic value of the males’ sexual performance and capacity as outlined by Huho *et al*. [40].

In order to perform unbiased competitiveness tests, the time of sexual maturation of both untreated and sterile males has to be similar so that neither one would have the opportunity to mate with females earlier. The genitalia rotation of males was overall not greatly affected by irradiation, although rotation was slightly slower for sterile males between 15 to 19 h post-emergence. Similarly, sterile males inseminated fewer females during the first 20 h but this difference was no longer visible after 25 h post-emergence. However, this difference observed between untreated and sterile males is only of concern for the establishment of competitiveness tests protocols.

Assuming that 40 Gy-irradiated males were equally competitive with untreated males, the fertility of the caged population should have averaged 49% (as the fertility of wild and sterile control males was 93 and 5% respectively). However, sterile one-day-old males had a low competitiveness index when competing in an equal ratio with wild males, and the wild female population fertility was only reduced with 10%. In Italy, competitiveness studies on *Ae. albopictus* indicated a good performance of sterile males irradiated at 30 Gy with a competitiveness index equal to 1.00±0.66; a high variability among replicates and between years was however reported [30]. A different strain, experimental setting and the diverse environmental conditions might affect the behaviour and survival of the mosquitoes, and could explain the differences observed between this study and ours. We observed that maintaining males for five days after emergence in the laboratory before the release greatly improved their competitiveness, and allowed the decrease of the semi-field cage wild population’s fertility to 62%, which indicated a nearly equal participation of both groups of males for the inseminations of females. The age of females differed between the experimental tests 1 and 2, as they were the same age as the males. However, females are already fully receptive to mating when two-days-old, and the receptivity to males and ability to retain semen should not differ with age as mentioned by Spielman et al. [41] for *Ae. aegypti* females. This almost four-fold improvement of the competitiveness value of the five-day-old males does not seem to be due to an increase of intrinsic male mating ability since, in laboratory conditions, a difference in male age did not affect the number of females inseminated. The time spent with an easily reachable sugar source in the insectarium during the pre-release period could have increased males nutritional status and thus improved some of their traits such as survival and flight capacity. A similar age effect on the mating competitiveness of sterile males *Culex pipiens fatigans* Wiedemann was reported by Krishnamurthy et al. [42] who observed that 36–60 h old males from a highly sterile male-linked translocated strain competed almost equally, in semi-field cages, against same age males from an untreated strain, whereas 12–36 h old males had a reduced competitiveness. Similarly, 36–60 h old chemosterilized *Ae. aegypti* males were competing better, in semi-field, against wild males than did 7–8 days-old males [31].We reported that a five-fold ratio of sterile to wild males allowed a reduction of almost 50% of the wild females natural fertility, suggesting that a 10-fold ratio could bring total sterility in a wild population and continuous releases might have a rapid efficient impact on the reduction of vectors density in the field. Releases of sterile males were usually performed with “over flooding ratios” so that the impact on the wild population would be faster [43], [44]. Laboratory studies on radiosterilized male *An. quadrimaculatus* Say allowed a reduction of 80% of the fertility of wild females when released in a ratio higher than 6∶1:1 (sterile males : wild males : wild females), but no reduction was observed at a ratio equal or less than 4∶1:1 [45]. A reduction of 95% in the fertility was possible in laboratory experiments with irradiated males *An. pharaoensis* Theobald competing in a ratio 10∶1:1 [46]. More recently, an average 5∶1 overflooding ratio of engineered sterile male *Ae. aegypti* occasioned an 80% reduction of a wild local population in the Cayman Islands over a 23-week period [47].

The results of this study suggest that a 5∶1 or higher sterile to wild male ratio should be combined with a pre-release period in an insectary to ensure the efficiency of a sterile male *Ae. albopictus* release. The *Ae. albopictus* density on Reunion Island is high and covers a wide range of habitats; although the ravines may be less easily accessible, releases near habitation gardens and parks should be straightforward. Releases at the pupal stage are often considered as more convenient, but it may be conceivable to use the emergence cage where the sterile males would be maintained during the pre-release period to bring them to the various release areas. Provided suitable aerial release systems can be developed and the surface of the treated area is large enough, aerial releases would ensure a cost-effective area-wide coverage. Furthermore, if the release at the adult stage is selected, it might then be of interest to irradiate males as adult in order to reduce the radiation induced somatic damages and thus improve their competitiveness [8]. The pre-release period may turn out to be an important cost factor in a mass-rearing facility; further studies should determine the minimal period required before release. The balance between sterility level and competitiveness of the sterile males is a major question for such programmes [48]. As in this study we chose a lower radiation dose ensuring a better competitiveness but not a complete sterility, the question remains whether the use of 5% fertile males is conceivable for a field release?

A reduction of competitiveness of radio-sterilized males is the key argument put forward to support a transgenic approach over classical SIT [49]. However, we showed that the effect of irradiation could be counteracted by adapting the release process, and does not prevent accomplishing an efficient reduction of an *Ae. albopictus* population’s fertility. In the native habitat, the competitiveness of the released sterile males will also depend on the effect of rearing and handling, the location of the release sites and the distribution of the wild mosquitoes [50]; a field trial is therefore now desirable to put sterile males to the test in conditions where they would also have to find sugar sources, locate female mates, and face predation risks.
